# New Mode of Treatment for Delayed or Non-Union of a Fractured Humerus

**Published:** 1854-08

**Authors:** Frank H. Hamilton


ART. II.—*New Mode of Treatment for Delayed or Non-Union of a Fractured Humerus.* Read before the Medical Society of the County of Erie. By Frank H. Hamilton, M. D.

It has been observed by surgeons that non-union results more frequently after fractures of the shaft of the humerus, than after fractures of the shaft of any other bone. This observation is confirmed by my own researches.

Comparing the humerus with the femur, between which, above all others, the circumstances of form, situation, &c., are most nearly parallel, and in both of which non-union is said to be relatively frequent, I find that of forty-nine fractures of the humerus, four occurred through the surgical neck, twelve through the condyles and twenty-nine through the shaft. In one of the twenty-nine, the patient survived the accident only a few days. In four of the remaining twenty-eight, union had not occurred after the lapse of six months, and in many more was it delayed considerably beyond the usual time. Two of the four were simple fractures, and occurred near the middle of the humerus; the third was compound, and occurred near the middle also; the fourth was compound, and occurred near the condyles.

This analysis supplies us, therefore, with four cases of non-union, from a table of twenty-eight cases of fractures through the shaft.

Of eighty-seven fractures of the femur, twenty occurred through the neck, one through the trocanter major, and one through the condyles. The remaining sixty-five occurred through the shaft and generally near the middle, and in not one case was the union delayed beyond six months.

To make the comparison more complete, I must add that of the twenty-eight fractures of the shaft of the humerus, six were compound; and of the sixty-five fractures of the shaft of the femur, six were either compound, comminuted, or both compound and comminuted. The six compound fractures of the shaft of the humerus, furnished two cases of non-union. The six cases of either compound or comminuted, or compound and comminuted fractures of the femur, furnished no case of non-union.

I beg to suggest to the Society what seems to me to be the true explanation of these facts.

It is the universal practice, so far as I know, in dressing fractures of the humerus, to place the forearm at right angles with the arm. Within a few days, and generally, I think, within a few hours, after the arm and forearm are placed in this position, a rigidity of the muscles and other structures has ensued, and to such a degree, that if the splints and sling are completely removed, the elbow will remain flexed and firm; nor will it be easy to straighten it. A temporary false anchylosis has occurred, and instead of motion at the elbow joint, when the forearm is attempted to be straightened upon the arm, there is only motion at the seat of fracture. It will thus happen that every upward and downward movement of the forearm will inflict motion upon the fracture, and inasmuch as the elbow has become the pivot, the motion at the upper end of the lower fragment will be the greater in proportion to the distance of the fracture from the elbow joint.

No doubt it is intended that the dressings shall prevent all motion of the forearm upon the arm; but I fear that they cannot always be made to do this. I believe it is never done when the dressing is made without angular splints, nor is it by any means certain that it will be accomplished when such splints are used. The weight of the forearm is such when placed at right angles with the arm and encumbered with splints and bandages, that even when supported by a sling, it settles heavily forwards, and compels the arm dressings to loosen themselves from the arm in front of the point of fracture, and to indent themselves in the skin and flesh behind. By these means the upper end of the lower fragment is tilted forward. If the forearm should continue to drag upon the sling, nothing but a permanent forward displacement would probably result. The bones might unite, yet with a deformity.

But the weight of the forearm under these circumstances is not uniform, nor do I see how it can be made so. It is to the sling that we must trust mainly to accomplish this important indication. But you have all noticed that the tension or relaxation of the sling depends upon the attitude of the body, whether standing or sitting—upon the erection or inclination of the head—upon the motions of the shoulders, and in no inconsiderable degree upon the actions of respiration. Nor does the patient himself cease to add to these conditions by lifting the forearm with his opposite hand whenever provoked to it by a sense of fatigue.

This difficulty of maintaining quiet apposition of the fragments while the arm is in this position, at whatever point the arm may be broken, becomes more and more serious as we depart from the elbow joint, and would be at its maximum at the extreme upper end of the humerus, were it not that here a mass of muscles, investing and adhering to the bone, in some measure obviates the difficulty. Its true maximum is therefore near the middle, where there is less muscular investment, and where, on the one hand, the fracture is sufficiently remote from the pivot or fulcrum to have the motion of the upper end of the lower fragment multiplied through a long arm, while on the other hand it is sufficiently near to the armpit and shoulder to prevent the upper portion of the splint and arm dressings from obtaining a secure grasp upon the lower end of the upper fragment.

It must not be overlooked that the motion of which we speak belongs exclusively to the lower fragment, and that it is always in the same plane, forwards and backwards; but especially that it is not a motion upon the fracture as upon a pivot, but a motion of one fragment to and from its fellow. This circumstance I regard as important to a right appreciation of the difficulty. Motion, alone, I am fully convinced, does not so often prevent union as surgeons have generally believed. It is exceedingly rare to see a case of non-union of the clavicle. Of forty-seven cases of fracture of the clavicle which have come under my observation, and in by far the greater majority of which considerable overlapping and consequent deformity has resulted—of this number only one has resulted in non-union, and in this instance no treatment whatever was practiced, but from the time of the accident the patient continued to labor in the fields and hold the plow as if nothing had occurred. I have therefore seen no case of non-union of the clavicle where a surgeon has treated the accident. Indeed, what is most remarkable, its union is more speedy, usually, than that of any other bone in the body, of the same size. Yet to prevent motion of the fragments in a case of fractured clavicle with complete separation and displacement, except where the fracture is near one of the extremities of the bone, I have always found wholly impracticable. Whatever bandages or apparatus I have applied, I have still seen always that the fragments would move freely upon each other at each act of inspiration and expiration, and at almost every motion of the head, body or upper extremities. It is probable, gentlemen, that you have made the same observation.

From this and many similar facts I have been led to suspect, for a long time, that motion has had less to do with non-union than was generally believed.

I find, however, no difficulty in reconciling this suspicion with my doctrine in reference to the case in question; and it is precisely because, as I have already explained, the motion, in case of a fractured humerus, dressed in the usual manner, is peculiar. In a fracture of the clavicle through its middle third, (its usual situation,) the motion is upon the point of fracture as upon a pivot; although, therefore, the motion is almost incessant, it does not essentially, if at all, disturb the adhesive process. The same is true in nearly all other fractures. The fragments move only upon themselves, and not to and from each other. I know of no complete exception but in the case now under consideration.

Aside of any speculation, the facts are easily verified by a personal examination of the patients during the first or second week of treatment, or at any time before union has occurred, both in fractures of the humerus and clavicle. The latter is always sufficiently exposed to permit you to see what occurs, and as soon as the swelling has a little subsided in the former case, you will have no difficulty in feeling the motion outside of the dressings, or perhaps in introducing the finger under the dressings sufficiently far to reach the point of fracture. I believe you will not fail to recognize the difference in the motion between the two cases.

Such, gentlemen, is the explanation which I wish to offer for the relative frequency of this very serious accident—non-union of the humerus.

I know of no other circumstance or condition in which this bone is peculiar, and which therefore might be invoked as an explanation. Overlapping of the bones, the reason assigned by some writers, is not sufficient, since it is not peculiar. The same occurs much oftener, and to a much greater extent, in fractures of the femur, and equally as often in fractures of the clavicle; yet in neither case are these results so frequent. Nor can it be due to the action of the deltoid or of any other particular muscles about the arm, whether the fracture be below or above their insertions, since similar muscles, with similar attachments on the femur and on the clavicle, tending always powerfully to the separation of the fragments, occasion only deformity, but not non-union.

If I am correct in my views, we shall be able sometimes to consummate union of a fractured humerus where it is delayed, by straightening the forearm upon the arm, and confining them to this position. A straight splint, extending from the top of the shoulder to the hand, made of some firm but moulding material, and made fast with rollers, will secure the requisite immobility to the fracture. The weight of the forearm and hand will only tend to keep the fragments in place, and if the splint and bandages are sufficiently tight, the motion occasioned by swinging the hand and forearm will be conveyed almost entirely to the shoulder joint. Very little motion indeed, can in this posture be communicated to the fragments, and what little is thus communicated, is a motion which experience has elsewhere shown not disturbing or pernicious, but a motion only upon the ends of the fragments as upon a pivot.

I do not fail to notice that this position has serious objections, and that it is liable to inconveniences which must always, probably, prevent its being adopted as the usual plan of treatment for fractured arms. It is more inconvenient to get up and lie down, or even to sit down, in this position of the arm; and the hand is liable to swell. But I shall not be surprised to learn that experience will prove these objections to have less weight than we are now disposed to give them. Remember, the practice is yet untried—if I except the case which I am about to relate, and in which case, I am frank to say, these objections scarcely existed. The swelling of the hand was trivial, and only continued through the first fortnight, and the patient never spoke of the inconvenience of getting up or sitting down, or even of lying down.

The following is the case to which I have just referred.

Michael Mahar, laborer, æt. 35, broke his left humerus just below its middle, Dec. 14, 1853. The arm was dressed by a skillful surgeon in Canada West, and who is well known to me as exceedingly "clever." After a few days from the time of the accident, "the starch bandage was put on as tight as it could be borne, and brought down on the forearm so as to confine the motions of the elbow joint."

Six weeks after the injury, Jan. 29, 1854, Mahar applied to me at the hospital. No union had occurred. The motion between the fragments was very free, so that they passed each other with an audible click. There was little or no swelling or soreness. In short, every thing indicated that union was not likely to occur without operative interference. The elbow was completely anchylosed. His health was unimpaired.

I explained to my students what seemed to me to be the cause of the delayed union, and declared to them that I did not intend to attempt to re-establish adhesive action until I had straightened the arm. They had just witnessed the failure of a precisely similar case in which I had made the attempt without straightening the arm and without success.

Feb. 6, 1854. I had succeeded in making the arm nearly straight. I now punctured the upper end of the lower fragment with a small steel instrument, and as well as I was able, thrust it between the fragments.

Assisted by Dr. Boardman, I then applied a gutta percha splint from the top of the shoulder to the fingers, moulding it carefully to the whole of the back and sides of the limb, and securing it firmly with a paste roller.

March 4th. (Not quite four weeks after the application of the splint,) I opened the dressings for the second time, and carefully renewed them. A slight motion was yet perceptible between the fragments.

March 18th. I opened the dressings for the third time, and found the union complete. This was within less than forty days.

The patient was now dismissed. On the 29th of April following the bone was refractured. Mahar had been assisting to load the "tender" to a locomotive. While the train was just getting in motion he was hanging to the tender by his sound arm when another laborer seized upon his broken arm to keep himself upon the car, and with a violent and sudden pull wrenched him from the tender and reproduced the fracture.

The next morning I applied the dressings as before, and did not remove it during three weeks, at the end of this time the union was again complete. The splint was, however, re-applied and has been continued to this time—a period of about six weeks.

